# Short-Term High-Fat Diet (HFD) Induced Anxiety-Like Behaviors and Cognitive Impairment Are Improved with Treatment by Glyburide

**DOI:** 10.3389/fnbeh.2016.00156

**Published:** 2016-08-11

**Authors:** Stephen J. Gainey, Kristin A. Kwakwa, Julie K. Bray, Melissa M. Pillote, Vincent L. Tir, Albert E. Towers, Gregory G. Freund

**Affiliations:** ^1^Department of Animal Sciences, University of IllinoisUrbana, IL, USA; ^2^Department of Pathology, Program in Integrative Immunology and Behavior, University of Illinois College of MedicineUrbana, IL, USA; ^3^Department of Nutritional Sciences, University of IllinoisUrbana, IL, USA

**Keywords:** glyburide, anxiety, cognition, high-fat diet, oxidative stress

## Abstract

Obesity-associated comorbidities such as cognitive impairment and anxiety are increasing public health burdens that have gained prevalence in children. To better understand the impact of childhood obesity on brain function, mice were fed with a high-fat diet (HFD) from weaning for 1, 3 or 6 weeks. When compared to low-fat diet (LFD)-fed mice (LFD-mice), HFD-fed mice (HFD-mice) had impaired novel object recognition (NOR) after 1 week. After 3 weeks, HFD-mice had impaired NOR and object location recognition (OLR). Additionally, these mice displayed anxiety-like behavior by measure of both the open-field and elevated zero maze (EZM) testing. At 6 weeks, HFD-mice were comparable to LFD-mice in NOR, open-field and EZM performance but they remained impaired during OLR testing. Glyburide, a second-generation sulfonylurea for the treatment of type 2 diabetes, was chosen as a countermeasure based on previous data exhibiting its potential as an anxiolytic. Interestingly, a single dose of glyburide corrected deficiencies in NOR and mitigated anxiety-like behaviors in mice fed with HFD-diet for 3-weeks. Taken together these results indicate that a HFD negatively impacts a subset of hippocampal-independent behaviors relatively rapidly, but such behaviors normalize with age. In contrast, impairment of hippocampal-sensitive memory takes longer to develop but persists. Since single-dose glyburide restores brain function in 3-week-old HFD-mice, drugs that block ATP-sensitive K^+^ (K_ATP_) channels may be of clinical relevance in the treatment of obesity-associated childhood cognitive issues and psychopathologies.

## Introduction

With over one billion overweight and obese individuals afflicted, world-wide overnutrition is a significant threat to human health (Kelly et al., 2008). Obesity is associated with increased susceptibility to various comorbidities like depression (Leckie and Withers, 1967; Luppino et al., 2010), type 2 diabetes (Wannamethee and Shaper, 1999), cardiovascular disease (Wannamethee et al., 1998), and cancer (Vainio and Bianchini, 2002). One of the root causes of obesity is attributed to ingestion of dietary fat (Lissner and Heitmann, 1995) which parallels the global permeation of a “western diet” (Cordain et al., 2005), where nearly 33% of total energy derives from fat. Predictably, extension of the obese phenotype into childhood is associated with added risks including diminished cognition and executive function (Liang et al., 2014). Additionally, obese children are susceptible to certain psychological complications like attention deficit hyperactivity disorder (ADHD), impulsivity, inattention and anxiety (Daniels et al., 2005; Kalarchian and Marcus, 2012). Current work demonstrates that childhood and adolescent obesity is disadvantageous, especially as psychological illness and/or cognitive impairment persists even when consumption of a high-fat diet (HFD) is well in the past (Wang et al., 2015). Given the projected prevalence of childhood obesity in the next 30 years (Ogden et al., 2014) and the magnitude of associated co-morbidities, identifying therapeutics to address this accelerating health concern is crucial.

Cognitive impairment and psychological abnormalities described in mouse models of diet-induced obesity (DIO; Buettner et al., 2012) are often connected to a reduction in molecules associated with neurogenesis and/or learning/memory such as brain derived neurotrophic factor (BDNF; Molteni et al., 2002), dopamine (DA; Kaczmarczyk et al., 2013) and inflammatory bioactives (Pistell et al., 2010). Recent studies demonstrate that DIO-associated brain dysfunction is not solely attributable to these mechanisms because hippocampal-based memory impaired by dietary fat occurs without significant change in BDNF (Heyward et al., 2013). Along similar lines, prolonged HFD-feeding does not upregulate brain-based IL-1β, TNF-α or IL-6 mRNA, even though sickness-like behaviors suggestive of brain inflammation are observed (Lavin et al., 2011). In juvenile animals, however, a HFD can impact working through pathways tied to glucocorticoids, neurogenesis and leptin (Boitard et al., 2012, 2014, 2015; Valladolid-Acebes et al., 2013), and it was found that this exposure impacted cognition in adulthood (Boitard et al., 2012). Although the brain-based complications of obesity are phenotypically well-described in animals, pharmacologic interventions that overcome such morbidities that are easily translated into humans are lacking.

The second-generation sulfonylurea, glyburide, has long been used in the treatment of type 2 diabetes (Kolterman et al., 1984; Groop, 1992). Glyburide inhibits sulfonylurea receptor 1 (Sur1) preventing K_ATP_ channel function (Ashcroft, 2005). It is this action on pancreatic β-cells that results in insulin release and improved blood glucose homeostasis (Niki et al., 1989; Zini et al., 1991). In turn, glyburide shows promise as is a promising brain therapeutic due to its ability to cross the blood-brain barrier (Simard et al., 2012). Since it has been explored as a countermeasure for traumatic brain injury (TBI), stroke, and spinal cord injury, it may be a suitable agent for other injuries and diseases as well (Kunte et al., 2007; Simard et al., 2009a,b; Patel et al., 2010). While glyburide has been explored as a mitigating agent in Alzheimer’s disease (Lavretsky and Jarvik, 1992), neuroinflammation (Lamkanfi et al., 2009; Koh et al., 2011) and oxidative stress-associated brain injury (Nazaroglu et al., 2009), little is known about the impact of sulfonylureas on the mental function of the disease it was originally designed to treat. To determine whether the sulfonylurea, glyburide, positively impacts HFD-induced cognitive impairment and anxiety-like behaviors in young mice, such behaviors were examined in animals 1, 3 and 6 weeks post-weaning fed with a low-fat or HFD. Thus, this pre-clinical study examines the role of glyburide as a deployable countermeasure to combat brain dysfunction associated with the early-life ingestion of a HFD.

## Materials and Methods

### Animals

The use of animals was in accordance with the Institutional Animal Care and Use Committee (IACUC) approved protocols at the University of Illinois Urbana, IL, USA. C57BL/6J male mice (3–4 weeks old) were purchased from Jackson Laboratories (Bar Harbor, ME, USA). Mice were placed on experimental diet 1 week after (between 4–5 weeks of age prior to puberty) arrival to allow for acclimation. Mice were group-housed (8 per cage), unless otherwise noted, in shoebox cages (length 46.9 cm; width 25.4 cm; height 12.5 cm) and allowed free access to food and water. Housing temperature (72°F) and humidity (45–55%) were controlled as was a 12/12 h reversed dark-light cycle (light = 1000–2200 h). All behavioral and biochemical experiments were performed in the dark cycle and separate cohorts to eliminate repeated measures as a factor. Individual mice were used in a single behavioral test. Total number of mice used was 396.

### Diets, Weights, Blood Glucose

Mice were initially fed a standard chow of NIH-31 modified open formula (Teklad 7013, Madison, WI, USA) containing 18% calories from protein, 6.2% from fat and 45% from carbohydrates. Mice were then transferred to a feed of open source uniform-base diets, for respective studies, containing either 10% calories from fat (low-fat diet [LFD]; D12450B, Research Diets, New Brunswick, NJ, USA) or 60% calories from fat (HFD; D12492, Research Diets, New Brunswick, NJ, USA). Both diets provided 20% calories from protein. Mouse weight was recorded for the respective weeks using an Adventurer Pro digital scale (Ohaus, Parsippany, NJ, USA). Blood glucose testing results were recorded for the respective weeks by fasting mice for 12 h during their light cycle and sampling tail blood. For glyburide studies, blood glucose testing was conducted immediately post-behavior testing with *ad libitum* access to food. Glucose was quantified by using an AlphaTRAK blood glucose monitoring system (Abbott Laboratories, North Chicago, IL, USA).

### Food Intake

As previously described (York et al., 2012), mouse cohorts used to determine food intake were individually housed for up to 6 weeks. Food intake was calculated daily as the difference in weight of food in the feed bowl before and after removal from the food intake arena.

### Plasma Non-Esterified Fatty Acids (NEFAs)

As described previously (Moon et al., 2014), mice were euthanized and blood collected via cardiac puncture using BD Microtainer Tubes with Lithium Heparin (BD Diagnostics, Franklin Lakes, NJ, USA). Blood was centrifuged at 8000× g for 10 min at 4°C. Supernatant was collected and analyzed. The resultant plasma non-esterified fatty acids (NEFA) were measured on an AU 680 Chemistry System (Beckman Coulter, Brea, CA, USA) using an enzymatic colorimetric NEFA test kit (Wako, Richmond, VA, USA). This kit is designed to measure total NEFA levels excluding short-chain fatty acids.

### Injectables

Glyburide (6.6 mg/kg/mouse; Sigma-Aldrich, St. Louis, MO, USA) was administered IP as described (Chiu et al., 2014) immediately prior to novel object training for mice at 1, 3 and 6 weeks on diet. Mice were injected 4 h prior to elevated zero maze (EZM) measured in mice on diet for 3 weeks only.

### Novel Object Recognition (NOR)

Testing was performed as described in the studies by Chiu et al. (2012) and York et al. (2012). In brief, group housed mice were transferred to a shoebox-style training arena (26 cm × 48 cm × 21 cm) containing two identical objects (LEGO toys in distinct configurations) on one side of the arena. Mice were allowed to investigate the objects for 24 h with food provided *ad libitum*. After training, mice were returned to their home cage for 1 h. After the 1 h refractory period, subject mice were transferred to individual testing arenas, without food but with bedding, where they were presented with one familiar object and one novel object in a spatial location comparable to training. Mouse exploration was video recorded for 5 min and evaluated by using EthoVision XT 7 video tracking software (Noldus Information Technology, Leesburg, VA, USA). A discrimination index was used to determine cognition and calculated as the amount of time spent examining the novel object divided by the total time spent investigating both objects.

### Object Location Recognition (OLR)

Testing was performed as described (York et al., 2012) and was similar to novel object recognition (NOR) except that upon testing the subject mouse was re-exposed to two familiar objects (LEGO toys) where one was placed at the opposite end from training. Spatial clues were placed on the outside of the cage to assist spatial determination. As above, mouse exploration was video recorded for 5 min and evaluated by using EthoVision XT 7 video tracking software. A discrimination index was used to determine cognition and calculated as the amount of time spent examining the object in a novel location divided by the total time spent investigating both objects.

### Elevated Zero-Maze

Testing was performed as described in the study by York et al. (2012). In brief, group-housed mice were individually housed for 24 h. For testing, subject mice were individually placed within the high walls of an EZM (57.15 cm outer diameter, 6 cm track, 72 cm from the floor). The maze was composed of four quadrants with two areas having high walls (14 cm tall) and two areas without walls. Mouse exploration was video recorded for 5 min and evaluated by using EthoVision XT 7 video tracking software. Time spent in the open quadrants was defined as at least 50% of the body being outside of the high-walled areas.

### Open Field Test

As above, group-housed mice were individual housed for 24 h. Mice were individually tested by placing subject mice in a lit novel open field arena (66 cm × 45.7 cm × 22.9 cm) generating a 9 cm shadow from respective side walls (York et al., 2012). Mouse exploration was video recorded for 5 min and evaluated by using EthoVision XT 7 video tracking software. Time spent in the open area was equated to time spent in the non-shadowed areas.

### Glutathione Assay

As previously described, PBS perfused brain regions were frozen in liquid nitrogen then freeze fractured (Kaczmarczyk et al., 2013) in reaction buffer containing 50 mM NaCl (Fisher Scientific, Fair Lawn, NJ, USA), 1 mM EDTA, 50 mM HEPES, pH 7.0 (USB Corporation, Cleveland, OH, USA) using the TissueLyser II (Qiagen, Valencia, CA, USA) at a rotational frequency of 30 s^−1^ for 2 min. Lysates were centrifuged at 10,000× g for 15 min at 4°C and the supernatant recovered. The supernatant was deproteinated with an equal volume of metaphosphoric acid (Sigma-Aldrich, St. Louis, MO, USA) and vortexing. Samples were re-centrifuged at 8000× g for 5 min. Supernatant and pellets were saved. Glutathione, both reduced and oxidized, was determined using the Glutathione Assay Kit (Cayman Chemical, Ann Arbor, Michigan) following the manufacturer’s instructions. Glutathione (GSH) and glutathione disulfide (GSSG) were quantified using an ELx800 Absorbance Microplate Reader (BioTek Instrument, Winooski, VT, USA) at 405 nm in 5 min intervals for 30 min. Protein precipitates were eluted with reaction buffer and quantified using the DC Protein Assay (Bio-Rad, Hercules, CA, USA).

### Quantitative PCR (qPCR)

Brain regions were dissected from PBS perfused whole brains and RNA isolated (York et al., 2012). RNA was reverse transcribed using the High-Capacity cDNA Reverse Transcription Kit (PN 4368813; Applied Biosystems, Foster City, CA, USA). The TaqMan Gene Expression primers used were: IL1R2 (Mm00439622_m1), BDNF (MM01334042_m1), Arc (Mm01204954_g1), iNOS (Mm00440502_m1), eNOS (Mm00435217_m1), casp1 (Mm00438023_m1), TXNIP (Mm01265659_g1), and superoxide dismutase (SOD1; Mm01344233_g1). Quantitative PCR (qPCR) was performed on a 7900 HT Fast Real-Time PCR System (Applied Biosystems, Foster City, CA, USA) using TaqMan Universal PCR Master Mix (Applied Biosystems, Foster City, CA, USA). To compare gene expression, a parallel amplification of endogenous RPS3 (Mm00656272_m1) was performed. Reactions with no reverse transcription and no template were included as negative controls. Relative quantitative evaluation of target gene to RPS3 was performed by comparing the values of ΔCts, where Ct is the threshold concentration.

### Statistical Analysis

Data analysis was conducted using Sigma Plot11.2 (Systat Software, Chicago, IL, USA). Body weight, fasting blood glucose (FBG), plasma NEFA, food intake, NOR and object location recognition (OLR) total investigation, EZM performance, open field testing (OFT), and PCR analysis used one-way analysis of variance (ANOVA) to define the main effects followed by Tukey adjustment. All experiments with NOR and OLR discrimination index analyzed using a one-sample *t*-test comparing novel object preference to chance level of 0.5. The Kruskal-Wallis one-way ANOVA on ranks was used for GSH:GSSG ratio for analyzing the main effect of diet and treatment in glyburide experiment, due to variance within groups. Glyburide experiments for blood glucose were analyzed by two-way ANOVA and time spent in open of EZM and closed arm entries by one-way ANOVA test to determine the main effects of diet and treatment followed by a Tukey adjustment. Statistical significance was assumed at *p* < 0.05 and all data are presented as means ± SEM.

## Results

### HFD Feeding for 3 Weeks Increases Body Weight and Blood Glucose Without Impacting Plasma NEFA Concentrations, Food Ingestion, and Inflammatory Gene Expression

To delineate the physiologic impact of the diets administered, the aforementioned biometrics were examined after 1, 3 and 6 weeks of feeding. When HFD-mice were compared to LFD-mice at 3 and 6 weeks post feeding, there was a 30% and 42% increase, respectively, in FBG levels. These findings correlated with a 13.4% and 28.4% rise in body weight, respectively (see Table 1). Interestingly, food intake was comparable in HFD-mice and LFD-mice, but this resulted in a 37%, 28%, and 14% increase in calories ingested in HFD-mice after 1, 3 and 6 weeks of feeding, respectively (see Table 1). Since HFD-associated brain-based inflammation is implicated in cognitive impairment, biomarkers of pro-inflammation were examined (Table 2). Interestingly, only hippocampal iNOS gene transcripts in HFD-mice showed an upregulation (99% vs. LFD-mice).

**Table 1:** **Body weight (g), blood glucose (mg/dL), plasma NEFA (mEq/L), food intake (g/d), and energy intake (kcal/g/d) of mice fed with LFD or HFD**.

	Time on diet					
	1 week		3 week		6 week	
Diet	LFD	HFD	LFD	HFD	LFD	HFD
Body weight	17.9 ± 0.4^a^	19.3 ± 0.9^b^	20.9 ± 0.4^a^	23.7 ± 0.5^b^	22.5 ± 0.5^a^	28.9 ± 0.5^b^
FBG	129.9 ± 8.2	164.1 ± 17.8	144.0 ± 10.1^a^	187.1 ± 6.9^b^	146.3 ± 16.2^a^	208.0 ± 16.0^b^
Plasma NEFA	0.784 ± 0.03	0.662 ± 0.23	0.633 ± 0.06	0.742 ± 0.04	0.638 ± 0.87	0.839 ± 0.10
Food intake	3.20 ± 0.6	3.23 ± 0.08	3.17 ± 0.17	2.98 ± 0.15	3.42 ± 0.7	2.88 ± 0.12
Energy intake	12.31 ± 0.18^a^	16.92 ± 0.41^b^	12.21 ± 0.18^a^	15.59 ± 0.41^b^	13.2 ± 0.18^a^	15.1 ± 0.41^b^

*Results are expressed as mean ± SEM, n = 4–30 per group. One-way ANOVA revealed main effect of diet (P < 0.05). Letters within rows indicate significant differences*.

**Table 2:** **Impact of LFD or HFD feeding on gene expression in the hippocampus and amygdala after 3 weeks of feeding**.

	Amygdala		Hippocampus	
Gene	LFD	HFD	LFD	HFD
Arc3.1	1.000 ± 0.140	0.759 ± 0.436	1.000 ± 0.250	0.751 ± 0.216
iNOS	1.000 ± 0.157	0.783 ± 0.147	1.000 ± 0.177^a^	1.993 ± 0.314^b^
eNOS	1.000 ± 0.087	0.965 ± 0.144	1.000 ± 0.119	1.277 ± 0.088
IL1-R2	1.000 ± 0.124	0.879 ± 0.316	1.000 ± 0.103	0.981 ± 0.201
Casp1	1.000 ± 0.035	0.856 ± 0.152	1.000 ± 0.152	0.936 ± 0.068
TXNIP	1.000 ± 0.108	1.075 ± 0.034	1.000 ± 0.196	0.996 ± 0.158
SOD1	1.000 ± 0.121	0.811 ± 0.044	1.000 ± 0.126	1.114 ± 0.080
BDNF	1.000 ± 0.112	1.333 ± 0.130	1.000 ± 0.168	1.092 ± 0.246

*Results are expressed as relative fold change in mRNA expression (mRNA), means ± SEM; n = 4. Results within individual rows without a common superscript letter are significantly different. Letters within rows indicate significant main effect of diet*.

### HFD-Mice Develop Object Memory Impairment and Transient Anxiety-Like Behaviors

**Figure 1:**
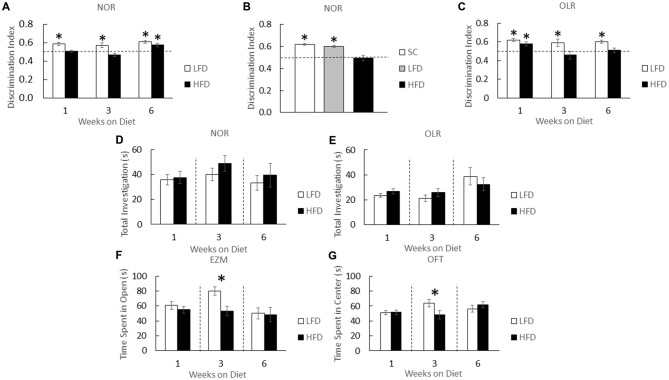
**High-fat diet (HFD) mice develop object memory impairment and transient anxiety-like behaviors.** HFD-mice (HFD), low-fat diet (LFD)-mice, and/or standard chow-mice (SC) underwent novel object recognition (NOR) at the times indicated (NOR) **(A)**, and at 1 week after diet **(B)**. HFD-mice (HFD) and LFD-mice (LFD) underwent object location recognition (OLR) testing at the times indicated **(C)**. Total time spent investigating both novel and familiar objects were determined for NOR **(D)** and OLR **(E)**. HFD-mice and LFD-mice were examined using elevated zero maze (EZM) **(F)** and the open field test (OFT) **(G)**. Discrimination index for NOR and OLR was defined as time spent exploring novelty divided by time spent investigating both objects. All results are expressed as means ± SEM; *n* = 4–16, values with an asterisk are significant at *p* < 0.05, using one-sample *t*-test with novel object preference compared with chance level of 0.5 and one-way analysis of variance (ANOVA).

HFD-mice and LFD-mice were examined after 1, 3 and 6 weeks of diet using a NOR task (Figure 1A). NOR is an effective measure of hippocampal-independent memory (Wan et al., 1999; Brown and Aggleton, 2001; McGaugh, 2004). At 1 and 3 weeks of diet, HFD-mice showed no preference for a novel object while LFD-mice showed preference for novel objects over that of the familiar ones (one-sample *t*-test: 1 week diet (LFD)- *P* < 0.001, 3 week diet (LFD)- *P* < 0.022; Figure 1A). After 6 weeks of diet, however, HFD-mice developed novel object preference which was similar to that of LFD-mice (one-sample *t*-test: 6 week diet (LFD)- *P* < 0.001, 6 week diet (HFD)- *P* = 0.005; Figure 1A). As an additional control, LFD-mice and chow-fed mice were compared. After 1 week of feeding, NOR performance in chow-fed and LFD-mice were comparable (one-sample *t*-test: SC- *P* = 0.002, LFD- *P* = 0.001; Figure 1B). HFD-mice and LFD-mice demonstrated similar total object exploration times (one-way ANOVA: 1 week diet- *F*_(1,22)_ = 0.08; *P* = 0.785, 3 week diet- *F*_(1,22)_ = 1.22; *P* = 0.281, 6 week diet- *F*_(1,22)_ = 0.317; *P* = 0.579; Figure 1D).

When hippocampal-memory was examined using OLR (Kesner et al., 1993; Broadbent et al., 2004; Jablonski et al., 2013) a persistent memory impairment was identified in HFD-mice compared to that of LFD-mice (Figure 1B). After 1 week of diet OLR was not impacted in HFD-mice (one-sample *t*-test: 1 week diet (LFD)- *P* < 0.001, 1 week diet (HFD)- *P* = 0.018; Figure 1C). However, after a period of 3 and 6 weeks of diet, HFD-mice lacked preference for the novel object whereas LFD-mice were able to distinguish between novel and familiar in the OLR task (one-sample *t*-test: 3 week diet (LFD)- *P* = 0.048, 6 week diet (LFD)- *P* < 0.001; Figure 1C). As above, diet did not impact combined object exploration (one-way ANOVA: 1 week diet- *F*_(1,12)_ = 1.870; *P* = 0.197, 3 week diet- *F*_(1,14)_ = 1.281; *P* = 0.277, 6 week diet- *F*_(1,22)_ = 0.565; *P* = 0.460; Figure 1E).

To explore the impact of a HFD on anxiety-like behavior mice were tested in an EZM. After 3 weeks of diet, time spent in the center of the EZM was reduced in HFD-mice compared to the time spent by LFD-mice (one-way ANOVA: *F*_(1,30)_ = 10.333; *P* = 0.003; Figure 1F). After 1 or 6 weeks of diet, no difference between LFD-mice and HFD-mice was observed. To further explore if a HFD engendered trait as opposed to state anxiety (Moon et al., 2015), the OFT was utilized (Prut and Belzung, 2003). HFD-mice spent a decreased amount of time in the open area of the OFT after 3 weeks of diet when compared to LFD-mice (one-way ANOVA: *F*_(1,23)_ = 4.446; *P* = 0.047; Figure 1G) and a similar amount of time in open after 1 or 6 weeks of diet.

### The GSH:GSSG Ratio in the Amygdala and Hippocampus is Reduced by an HFD

**Figure 2:**
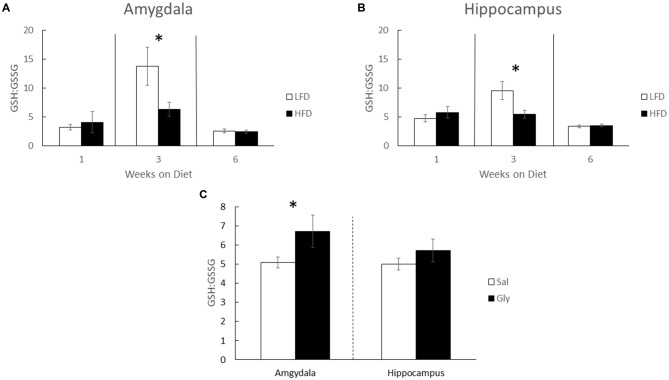
**The GSH:GSSG ratio in the amygdala and hippocampus is reduced by a HFD.** Glutathione (GSH) and glutathione disulfide (GSSG) were measured in HFD and LFD-mice and presented as GSH:GSSG in both the amygdala **(A)** and hippocampus **(B)**. GSH:GSSG ratios in amygdala and hippocampus of HFD-mice injected with glyburide **(C)**. Results are expressed as means ± SEM, *n* = 5–10, values with an asterisk are significant, *p* < 0.05 using Kruskal-Wallis one-way ANOVA on ranks.

Since antioxidant capacity can impact memory (Alzoubi et al., 2013; Xu et al., 2014) brain GSH and GSSG was examined. After 3 weeks of diet, there was a 54% and 43% increase in the GSH:GSSG ratios in the amygdala and hippocampus, respectively, when LFD-mice were compared to HFD-mice (Kruskal-Wallis one-way ANOVA: amygdala- *Q* = 2.935; *P* = 0.038; Figure 2A, hippocampus- *Q* = 2.449; *P* = 0.014; Figure 2B). GSH:GSSG ratios were not different in LFD-mice and HFD-mice after 1 and 6 weeks of diet. Glyburide, which is known to increase antioxidant capacity (Patel et al., 1987; Chugh et al., 2001), raised the GSH:GSSG ratio 32.3% in the amygdala, in HFD-mice after 3 weeks of diet (Kruskal-Wallis one-way ANOVA: Amygdala- *Q* = 2.748; *P* = 0.050; Figure 2C).

### Glyburide Reduces Anxiety-Like Behaviors Associated with a HFD After 3 Weeks of Diet

**Figure 3:**
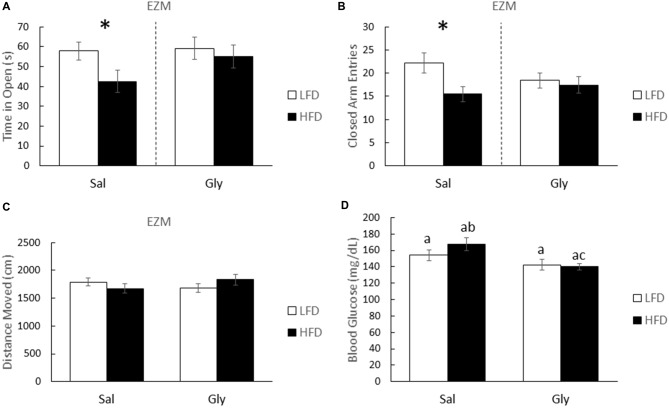
**Glyburide reduces anxiety-like behaviors associated with a HFD after 3 weeks of diet.** HFD and LFD-mice were administered either saline or glyburide and examined after 4 h in an EZM. Time in the open **(A)** and closed arms **(B)** are presented. Similarly treated HFD- and LFD-mice were examined for locomotion **(C)**, and post-behavior blood glucose **(D)**. All results are expressed as mean ± SEM; *n* = 10–18, values without a common subscript or asterisk are different (a vs. b, *p* < 0.05) with **(A,B)** analyzed using one-way ANOVA and **(C,D)** using two-way ANOVA.

After glyburide treatment, HFD-mice and LFD-mice spent equivalent times exploring the open and closed arms of the EZM (one-way ANOVA: Gly- *F*_(1,35)_ = 0.277; *P* = 0.602; Figure 3A; *F*_(1,35)_ = 0.166; *P* = 0.686; Figure 3B). Importantly, injection of saline did not prevent anxiety-like behaviors associated with a HFD (one-way ANOVA: Sal- *F*_(1,33)_ = 4.460; *P* = 0.043; Figure 3A; *F*_(1,33)_ = 6.264; *P* = 0.018; Figure 3B). HFD-mice and LFD-mice did not differ in distance moved in the EZM (see Figure 3C). As expected, *post hoc* analysis showed that glyburide resulted in a 16.5% reduction in non-FBG levels (two-way ANOVA: HFD (Sal vs. Gly)- *Q* = 4.506; *P* = 0.003; Figure 3D) and was associated with an overall drug effect (two-way ANOVA: Sal vs. Gly- *F*_(1,41)_ = 9.418; *P* = 0.004; Figure 3D).

### Memory is Improved by Glyburide in HFD-Mice After 3 Weeks of Diet

**Figure 4:**
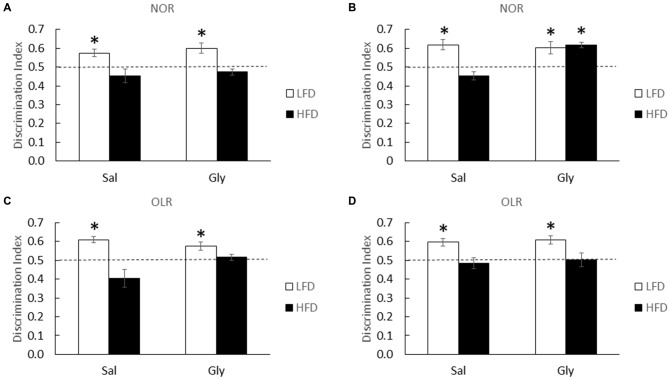
**Memory is improved by glyburide in HFD-mice after 3 weeks of diet.** HFD and LFD-mice were administered either saline or glyburide after a 1 week of diet and examined using NOR testing **(A)**. HFD- and LFD-mice were administered either saline or glyburide after a 3 weeks of diet and examined using NOR **(B)** and OLR **(C)** testing. HFD and LFD mice were administered either saline or glyburide after a 6 weeks of diet and examined using OLR **(D)** testing. Discrimination index was defined as time spent exploring novelty divided by time spent investigating both objects. Results are expressed as means ± SEM; *n* = 4–6, values with an asterisk are significant at *p* < 0.05, using one-sample *t*-test with novel object preference compared with chance level of 0.5.

At 1, 3 and 6 weeks of diet, HFD-mice were unable to discriminate novelty in the NOR and OLR tasks (one-sample *t*-test: 1 week NOR (LFD-Sal)- *P* = 0.013, 1 week (HFD-sal)- *P* = 0.249; Figure 4A; 3 week NOR (LFD-Sal)- *P* = 0.008, 3 week (HFD-Sal)- *P* = 0.089; Figure 4B; 3 week OLR (LFD-Sal)- *P* < 0.001, 3 week (HFD-Sal)- *P* = 0.145; Figure 4C; 6 week OLR (LFD-Sal)- *P* = 0.005, 6 week (HFD-Sal)- *P* = 0.644; Figure 4D). Interestingly, glyburide restored novelty preference in HFD-mice in the NOR task after 3 weeks of diet (one-sample *t*-test: 3 week NOR (LFD-Gly)- *P* = 0.025, (HFD-Gly)- *P* < 0.001; Figure 4B), but not at 1 or 6 weeks of diet (one-sample *t*-test: 1 week NOR (LFD-Gly)- *P* = 0.018, 1 week (HFD-Gly)- *P* = 0.165; Figure 4A; 6 week OLR (LFD-Gly)- *P* = 0.002, 6 week (HFD-Gly)- *P* = 0.915; Figure 4D) and not for OLR (one-sample *t*-test: 3 week OLR (LFD-Gly)- P 0.014, 3 week (HFD-Gly)- *P* = 0.351; Figure 4C).

## Discussion

Overweight children are predisposed to social and emotional complications tied to overnutrition including depression, low self-esteem and learning problems (Mellbin and Vuille, 1989; Daniels et al., 2005). Origination of these learning problems is associated with inferior social skills (Dietz, 1998) and anxiety (Williams, 2001). In addition, controversy exists as to whether the overweight/obese phenotype is a root cause of childhood social and emotional problems or a sequela of resultant bullying (Eisenberg et al., 2003). In juvenile mice, a short-term (1-week) HFD feeding impairs behavior as we have previously shown (Kaczmarczyk et al., 2013) and as shown here (Figure 1A). Consistently, impaired NOR appears associated with short-term overnutrition, as opposed to impaired OLR which takes longer to manifest (3 weeks; Figure 1B). Interestingly, this early impairment in NOR is not associated with FBG or plasma NEFAs but does positively correlate with energy intake (Table 1). These findings support the contention that learning problems associated with overnutrition are not entirely linked to the overweight/obese body type and its negative perception by industrialized peoples (Murray et al., 2009; Holloway et al., 2011).

Another important finding is that impairment of OLR is longer lasting than impairment of NOR (Figure 1). The advantage of using NOR and OLR for memory testing is that they are easy to perform, involve a similar paradigm but test different types of memory. NOR is much more hippocampal-independent than is OLR (Wan et al., 1999; Brown and Aggleton, 2001; McGaugh, 2004). In contrast, OLR is more dependent on spatial memory and is, thus, more hippocampal-sensitive (Moses et al., 2005). Our findings are supported by recent work which shows that 23 weeks of HFD feeding in mice impairs OLR but not NOR (Heyward et al., 2013). In addition, we previously found that NOR was intact in HFD-mice on diet for 10–12 weeks (Lavin et al., 2011). Taken together, the clinical significance of these works is their relationship to dementia and type 3 diabetes (T3D; de la Monte and Wands, 2008).

In T3D, lack of brain-based insulin production or the presence of brain-based insulin resistance (Biessels et al., 1998; Lannert and Hoyer, 1998; Kodl and Seaquist, 2008) precipitates memory dysfunction with symptomatology that overlaps that of Alzheimer’s disease (Gasparini et al., 2001; Steen et al., 2005; Deng et al., 2009). Insulin is required for new memory creation by facilitating synaptic plasticity (van der Heide et al., 2006) and HFD-induced insulin/IGF-1 resistance (Spielman et al., 2014) which appears important to T3D (Watson and Craft, 2004; Vardy et al., 2007), especially in the hippocampus (McNay et al., 2010; Grillo et al., 2015). Given that HFD-mice have an elevation in FBG (Table 1), it is not surprising that impairment in hippocampal-dependent memory would be coincident (Figure 1). Furthermore, HFD-induced elevations in FBG are caused by insulin resistance (Hirosumi et al., 2002). What was not anticipated is the rapidity by which a HFD impairs hippocampal memory. Although Beilharz et al. (2014) found a similar phenomenon in their study, they concluded that dietary sugar was the critical factor. Sugar availability (~6.7% sucrose) was low in the diets used here. Thus, the hippocampal memory impairment observed appears dependent on fat content not on the sugar content.

Previous work by André et al. (2014) showed cognitive impairment and anxiety-like behaviors in mice fed with HFD. In their study, the postulated mechanism was tied to pro-inflammatory cytokines and indoleamine 2, 3-dioxygenase (IDO) activity. In contrast, Kaczmarczyk et al. (2013) demonstrated no brain-based pro-inflammation in HFD-exposed mice and that IDO knockout mice were as susceptible to HFD-induced memory impairment as were the controls. A key difference between the studies of Kaczmarczyk et al. (2013) and André et al. (2014) was the length of diet which was marked shorter in the latter’s study. Finally, Del Rio et al. (2016) fed juvenile mice a short-term HFD resulting in cognitive deficits and an anti-depressive phenotype. Anxiety-like behaviors were not observed, but the diet that Del Rio et al. (2016) used contained 45% fat as opposed to the 60% used in this study.

Why a HFD impacts hippocampal-independent functions like NOR and anxiety-like behaviors (Figures 1A,D,E) rapidly and transiently is not clear. Previous work shows that oxidative stress is deleterious to brain function (Shukitt-Hale, 1999; Dröge and Schipper, 2007). Specifically, anxiety-like behaviors manifest in mice when the GSH:GSSG ratio is reduced and cytosolic reactive oxygen species are increased (Llorente-Folch et al., 2013). While Figure 2 shows a drop in the GSH:GSSG ratio in HFD-mice compared to LFD-mice, this result appears as a consequence of a HFD-induced suppression of a rise in the GSH:GSSG ratio. Interestingly, 7 weeks marks the approximate sexual maturation of male C57BL/6J mice (range 6–8 weeks; Fox and Witham, 1997). Thus, male sexual maturation appears to be accompanied by a spike in the brain GSH:GSSG ratio (Figure 2). Mechanistically, this observation may be tied to an increase in testosterone since brain GSH in mice (Atroshi et al., 1990) is augmented by its administration. Additionally, sexual maturation increases the brain-active antioxidant dehydroepiandrosterone (DHEA; Hopper and Yen, 1975; McIntosh and Berdanier, 1991; Aly et al., 2011), and DHEA favorably impacts the hippocampus in neurodegenerative diseases (Charalampopoulos et al., 2008). Glyburide, especially during uncontrolled diabetes, increased reduced glutathione (Chugh et al., 2001). Such findings lend credence to our results demonstrating that glyburide increased the GSH:GSSG ratio in the brain. However, this effect appears brain-region specific, when the amygdala and hippocampus are compared.

As Figures 3, 4 illustrate, an acute single dose of glyburide prevents HFD-induced memory impairment and anxiety-like behaviors indicating a role for this sulfonylurea in overnutrition-associated brain dysfunction in juvenile mice. Previously, we demonstrated that glyburide can block the activation of brain caspase-1 triggered by adenosine (Chiu et al., 2014) which is a key biologic in hypoxia-induced anterograde amnesia (Chiu et al., 2012). Since overnutrition is associated with endoplasmic reticulum (ER) stress (Mollica et al., 2011), it is theorized that the overnutrition-associated oxidative stress causes dysregulated purine metabolism (Al-Rubaye and Morad, 2013). Subsequent cellular release of ATP and its precursors rapidly increases the interstitial concentration of adenosine (Chiu and Freund, 2014) which through the A2A adenosine receptor triggers neuronal hyperpolarization in a K_ATP_ channel dependent manner (Popoli et al., 2002). Hence the relevance of adenosine to neurodegeneration and sleep (Portas et al., 1997; Stone, 2005) and the interest in caffeine and its derivatives as neuroprotectants and CNS stimulants (Schwarzschild et al., 2002; Barranco Quintana et al., 2007). Interestingly, a recent study identified the P2X7 receptor as a potential link between anxiety in rats and a HFD (Dutheil et al., 2016). This study, however, demonstrated HFD-induced brain inflammation as the potential mechanism. As noted, brain-based inflammation was not observed here likely due to our use of a short-term HFD feeding. Dutheil et al. (2016) fed rats an HFD for 16 weeks. Thus, different pathways are likely at play during the early (3 week) and late (12–16 week) manifestations of anxiety associated with a HFD.

Glyburide can also boost SOD and catalase activity (Nazaroglu et al., 2009) mitigating oxidative stress in a canonical fashion. However, due to its limited impact on blood glucose (Figure 3), this mechanism seems unlikely In HFD-mice fed with fat for at least 6 weeks, memory-impairment may be due to hyperglycemia and the role blood glucose has in elevating extracellular amyloid-β protein in the hippocampus (Macauley et al., 2015).

In summary, short-term HFD-feeding induces both cognitive impairment and anxiety-like behaviors which parallel symptoms seen in childhood obesity. These results coincide with a reduction in antioxidant capacity as exhibited by a suppressed GSH:GSSG ratio. Importantly, we demonstrated that administration of glyburide ameliorated hippocampal-independent brain function in HFD-mice. Unfortunately glyburide did not influence the hippocampal-sensitive memory task tested. Concordantly, glyburide increased the GSH:GSSG ratio in the amygdala but not in the hippocampus. Therefore, glyburide appears to selectively increase antioxidant capacity in the brain resulting in mitigation of hippocampal-independent impairments linked to a short term exposure to a HFD. These results suggest a unique use for glyburide in the prevention of anxiety and hippocampal-independent cognitive impairment. While the exact mechanism of action requires further study, antioxidant capacity appears to be an important glyburide target.

## Author Contributions

KAK, JKB, MMP, and VLT contirbuted to the acquisition, analysis, and interpretation of the behavioral and biochemical data. SJG contributed to the design, acquisition, analysis, and interpretation of the whole of this manuscript. AET contributed to the design, interpretation, and revision of all aspects of this manuscript. GGF contributed to the design, analysis and interpretation of all aspects of this manuscript. All authors worked on drafting and reviewing this manuscript, and approved the final version for publication.

## Conflict of Interest Statement

The authors declare that the research was conducted in the absence of any commercial or financial relationships that could be construed as a potential conflict of interest.

## Abbreviations

DA: dopamine
DHEA: dehydroepiandrosterone
DIO: diet induced obesity
DOPAC: 3, 4-dihydroxyphenylacetic acid
EZM: elevated zero maze
FBG: fasting blood glucose
GSH: glutathione
GSSG: glutathione disulfide
HFD: high fat diet
HFD-mice: high-fat diet mice
HVA: homovanillic acid
IDO: indoleamine 2, 3 deoxygenase
LFD: low fat diet
LFD-mice: low-fat diet mice
NEFA: non-esterified fatty-acids
NOR: novel object recognition
OFT: open field test
OLR: object location recognition
SOD: superoxide dismutase.
